# Tolfenpyrad displays *Francisella*-targeted antibiotic activity that requires an oxidative stress response regulator for sensitivity

**DOI:** 10.1128/spectrum.02713-23

**Published:** 2023-10-06

**Authors:** Ashley Clarke, Isabelle M. Llabona, Nimra Khalid, Danielle Hulvey, Alexis Irvin, Nicole Adams, Henry S. Heine, Aria Eshraghi

**Affiliations:** 1 Department of Infectious Diseases & Immunology, University of Florida, Gainesville, Florida, USA; 2 Institute for Therapeutic Innovation, University of Florida, Orlando, Florida, USA; 3 Emerging Pathogens Institute, University of Florida, Gainesville, Florida, USA; 4 Department of Oral Biology, University of Florida, Gainesville, Florida, USA; University of Manitoba, Winnipeg, Manitoba, Canada

**Keywords:** *Francisella*, tularemia, antibiotics, small molecule inhibitor, reactive oxygen species

## Abstract

*Francisella tularensis* is a highly infectious Gram-negative bacterial pathogen capable of animal-to-human transmission. Due to its remarkable pathogenicity and potential for widespread public health impact, *F. tularensis* is classified as a high-priority pathogen by the Centers for Disease Control and Prevention. The historical development of *Francisella* as a bioweapon, coupled with the ease of engineering antibiotic-resistant strains, has spurred interest in the discovery of novel antibiotics. A library of drug-like compounds was screened for their ability to inhibit the growth of *Francisella novicida*, a biosafety level-2 surrogate for *F. tularensis*. Tolfenpyrad, a pesticide without previously identified antibacterial activity, was among the most potent inhibitors. Tolfenpyrad appears to uniquely target *Francisella*, as related species were sensitive, whereas bacteria in other genera were resistant. To identify *Francisella* pathways conferring sensitivity to tolfenpyrad, whole-genome sequencing of tolfenpyrad-resistant isolates was performed. Point mutations in two genes conferred resistance to osrR, a transcriptional regulator, and nuoM, a subunit of the electron transport chain. OsrR point mutants displayed attenuated growth in interferon gamma (IFN-γ)-stimulated macrophages but not in untreated macrophages, indicating that OsrR plays a role in resistance to IFN-γ-stimulated clearance of intracellular bacteria. OsrR point mutants are hypersensitive to reactive oxygen species, suggesting a mechanism by which they are rapidly cleared from IFN-γ-treated macrophages. This work identifies a novel *Francisella*-specific antibiotic activity and the metabolic pathways targeted. Moreover, we provide amino acid-level details of a *Francisella* protein that governs sensitivity to free radicals.

**IMPORTANCE:**

*Francisella* species are highly pathogenic bacteria that pose a threat to global health security. These bacteria can be made resistant to antibiotics through facile methods, and we lack a safe and protective vaccine. Given their history of development as bioweapons, new treatment options must be developed to bolster public health preparedness. Here, we report that tolfenpyrad, a pesticide that is currently in use worldwide, effectively inhibits the growth of *Francisella*. This drug has an extensive history of use and a plethora of safety and toxicity data, making it a good candidate for development as an antibiotic. We identified mutations in *Francisella novicida* that confer resistance to tolfenpyrad and characterized a transcriptional regulator that is required for sensitivity to both tolfenpyrad and reactive oxygen species.

## INTRODUCTION

*Francisella tularensis* subspecies *tularensis* Schu S4 (*F. tularensis*) is a highly pathogenic, Gram-negative, γ-proteobacterium that causes tularemia in a diverse array of animals, including humans. *F. tularensis* is one of the most potent bacterial pathogens known, and its remarkable virulence is exemplified by a lethal dose below 10 viable bacteria that leads to respiratory failure, septicemia, and 30%–60% mortality if untreated (1 – 4). Tularemia is transmitted through multiple routes, including vectors, direct contact with infected animals, and the inhalation of aerosolized bacteria. Strains of *F. tularensis* have been previously developed as biological weapons with the intention of aerosolized dissemination (4, 5). The type and severity of tularemia depend on the route of transmission, and the most serious form of the disease is respiratory tularemia, which is acquired through inhalational exposure or the accumulation of bacteria in the lungs following dissemination from other routes. Symptoms of respiratory tularemia are nonspecific and include fever, chills, malaise, and cough, complicating the diagnosis (6).

*Francisella* species are intracellular pathogens that employ virulence factors in a complex and dynamic infection pathway. Upon entry into a mammalian host, they evade detection and clearance during a transient extracellular phase by producing an atypical lipopolysaccharide structure that is poorly recognized and secreting a capsule that confers resistance to complement-mediated lysis (7 – 9). Once phagocytosed by macrophages, a unique type VI secretion system (T6SS) exports toxins that block fusion of the *Francisella*-containing phagosome with lysosomes in order to resist degradation (10). T6SS-exported toxins also facilitate the egress of the bacterium into the cytosol, where they gain access to nutrients required for proliferation (11, 12). Macrophages secrete Th1-type cytokines, including interferon gamma (IFN-γ), which plays a central role in the response to intracellular *Francisella*. Priming macrophages with IFN-γ induces the production of reactive oxygen and nitrogen species upon detection of cytosolic *Francisella* (13). This respiratory burst is often an effective strategy to clear intracellular pathogens; however, *Francisella* has evolved multiple strategies to counteract these toxic radicals (14 – 17). Cellular infection concludes with host cell lysis to release infectious *Francisella* or direct bacterial translocation through the cell membrane into adjacent cells to cause tissue damage and eventual sepsis (18). These virulence factors make *Francisella* a highly successful pathogen, and therefore, we must devise new strategies to target and inhibit the growth of these bacteria.

Tularemia can be treated by standard antimicrobial therapy, and antibiotic resistance to frontline therapeutics has not been detected in clinical isolates; however, resistant strains can be readily generated with rudimentary methods and limited resources (19 – 24). Given these characteristics, *F. tularensis* is categorized as a “Category A” agent of concern by the United States Centers for Disease Control and Prevention. Since *F. tularensis* poses an ongoing threat to global health security, we must put significant effort toward elucidating the mechanisms it employs for pathogenesis so that we can develop countermeasures to bolster public health preparedness. The discovery of novel antibiotics poses a formidable challenge. The process is complex, lengthy, and expensive, and attrition of candidates is high due to numerous potential pitfalls on the road to approval. Drug repurposing is a strategy for discovering new activities in compounds that were originally developed for an unrelated therapeutic indication. This approach has several advantages, the most important of which is a lower likelihood of failure due to preexisting knowledge of drug characteristics (25).

In this study, we screened a library of existing drugs provided by Medicines for Malaria Venture (MMV) and found that an approved pesticide, tolfenpyrad, has a previously undiscovered antibacterial activity that blocks *Francisella* growth. Using a chemical genetic approach, we found mutations in two *Francisella* genes that confer resistance to tolfenpyrad: a subunit of the electron transport chain, NuoM, and a transcriptional regulator, OsrR. Furthermore, we present evidence that these amino acid residues in OsrR are required for intracellular bacterial growth by controlling the ability of the bacteria to tolerate reactive oxygen species. Finally, we identify key residues in related transcriptional regulators that may play a role in sensing stimuli and binding DNA. These findings are a basis for future studies on generating lead compounds that target *Francisella* and characterize the molecular details of a family of transcriptional regulators.

## RESULTS

### Discovery of *Francisella novicida*-inhibiting drugs

**FIG 1:**
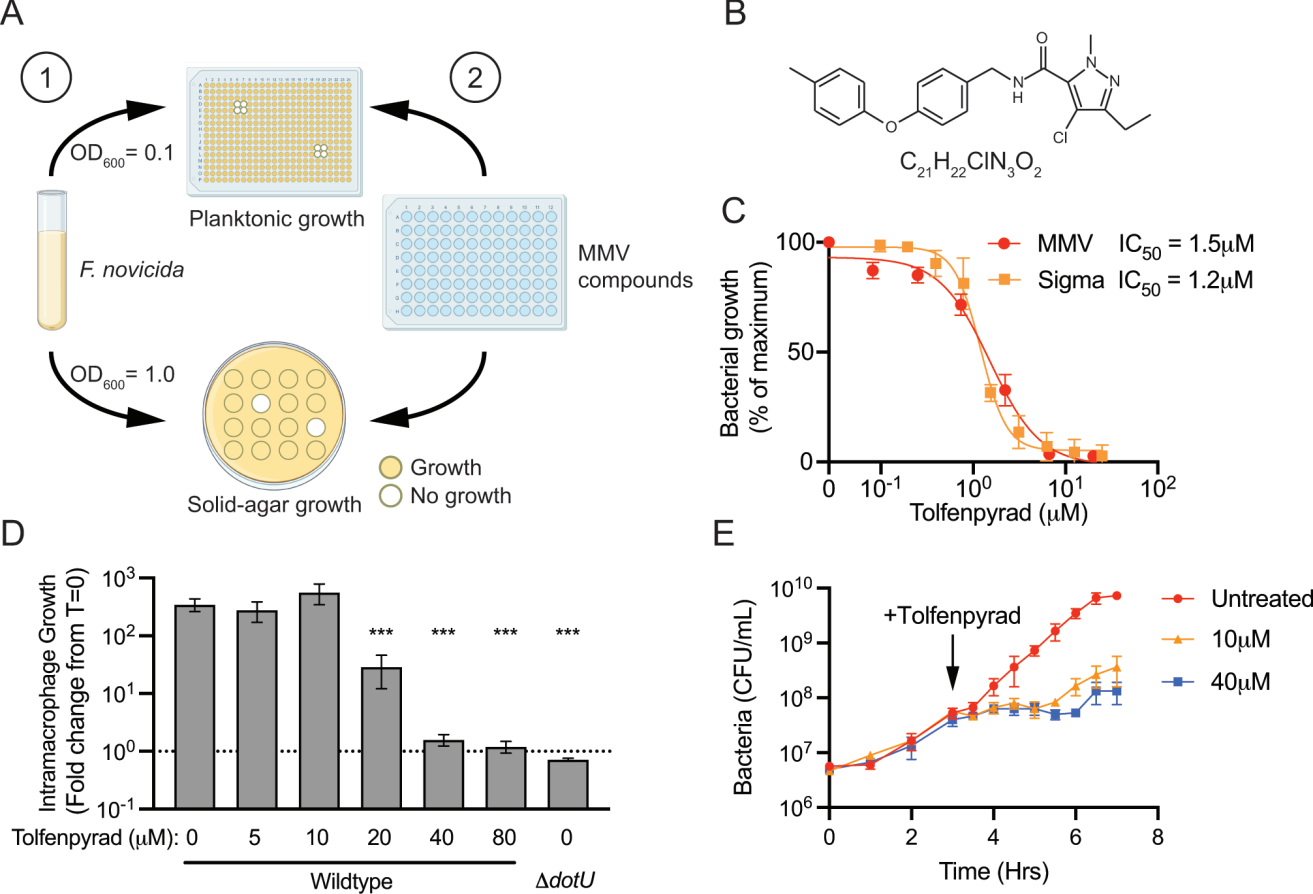
Screening the MMV compound libraries reveals a novel inhibitor of *F. novicida* growth. (**A**) Schematic of the two screening approaches utilized to screen for inhibitors of planktonic and solid-agar growth of *F. novicida*. A log-phase bacterial culture was used to inoculate either a 384-well plate or Petri dish containing tryptic soy broth + 0.1% cysteine or agar, followed by the addition of MMV compounds at 12.5 µM. Each compound was tested in quadruplicate on a single 384-well plate or in duplicate on two Petri dishes. (**B**) Chemical structure and formula of tolfenpyrad. (**C**) Dose-response curves and calculated IC_50_ of tolfenpyrad obtained from MMV and Sigma Aldrich. (**D**) Intramacrophage growth of wild-type *F. novicida* in the presence of tolfenpyrad and an avirulent type VI secretion system mutant (Δ*dotU*) within immortalized murine bone marrow-derived macrophages. The y-axis denotes the fold change from *T* = 0. ***P ≤ 0.0003. (**E**) Growth of *F. novicida in vitro* and addition of the indicated concentrations of tolfenpyrad at 3 h post inoculation (arrow).

The MMV compound collection is composed of the following three sub-libraries: the Pandemic Response Box, Pathogen Box, and COVID Box. In total, these libraries are composed of 935 unique compounds that target a variety of pathogens and have known cytotoxicity and pharmacokinetic properties (26). In our screening experiment, we utilized *F. tularensis* subsp. *novicida* U112 (*F. novicida*), a biosafety level-2 surrogate for *F. tularensis* that is not pathogenic to healthy humans but is 97% identical at the nucleotide level. We screened for the ability of these compounds to inhibit the growth of *F. novicida* in both liquid media and on solid agar at 12.5 µM, visually examining for the absence of turbidity and zones of clearing, respectively (Fig. 1A). These screens revealed 46 compounds that inhibited growth in liquid media and 34 compounds on agar, 23 of which blocked bacterial growth on both types of media (Table S1). Many of the 23 drugs are known antibiotics, confirming that the screen is sufficiently sensitive and robust to identify antibacterial compounds. Additionally, a recently characterized antibacterial identified in a *Vibrio cholerae* screen, MMV675968, inhibited *F. novicida* growth in our screen as well, extending the known spectrum of that drug and further validating our approach (27). Notably, one drug without a previously identified antibacterial activity, tolfenpyrad, blocked *F. novicida* growth. Tolfenpyrad was first reported as an insecticide in 1996 and approved for use in Japan in 2002 (Fig. 1B). Since then, it has been extensively used, including in the United States, India, China, and Brazil.

To validate and quantify the efficacy of tolfenpyrad to block *F. novicida* growth, we employed dose-response assays *in vitro* and in a cell culture model for infection. We first confirmed that the tolfenpyrad obtained from MMV is equally potent as that obtained from a second vendor (Sigma Aldrich) by measuring bacterial growth in a titration of tolfenpyrad from the two sources. At midlog phase, tolfenpyrad inhibits *F. novicida* growth with an IC_50_ of 1.2 µM (±0.1 µM) or 1.5 µM (±0.2 µM), depending upon the source (Fig. 1C). Based on these results, the potency of tolfenpyrad *in vitro* exceeds that of recently discovered antimicrobials that target *Francisella* (28, 29).

Tolfenpyrad exerts toxicity in insects through inhibition of cellular respiration. Across metazoans, the characteristics of plasma membranes differ extensively, including their permeability to drug-like compounds. Tolfenpyrad targets an intracellular protein in insects; however, it is unclear whether it can transverse the mammalian membrane to control *Francisella* growth within host cells. To determine if tolfenpyrad can block intracellular growth of *F. novicida in vivo*, a modified gentamycin protection assay was applied by using cultured immortalized murine bone marrow-derived macrophages (iBMDMs). These cells were briefly incubated with bacteria to allow for phagocytosis, followed by the clearance of extracellular bacteria with gentamycin treatment. After extensive washing, cells were treated with a titration of tolfenpyrad, and intracellular bacteria were enumerated immediately and 16 h post infection. Intracellular bacterial growth was attenuated in a dose-dependent fashion, with significant attenuation displayed at 20 µM. Bacterial growth was completely inhibited at 40 µM, similar to an avirulent mutant, Δ*dotU* (Fig. 1D). Substantial toxicity to iBMDM cells was only observed at 80 µM as judged by the MTT assay, suggesting that tolfenpyrad is effective *in vivo* by directly inhibiting the growth of *F. novicida*, not through toxicity of the host cells (Fig. S1).

Antibiotics are classified as bacteriostatic or bactericidal based on their ability to block bacterial growth or kill bacteria, respectively. To classify tolfenpyrad into one of these categories, we tested the mechanism by which it inhibits *F. novicida*. Bacterial growth was monitored, and either 10 or 40 µM tolfenpyrad was added at the early log phase (Fig. 1E). Enumeration of viable bacteria over time revealed that tolfenpyrad inhibits *F. novicida* growth but does not reduce viability at either concentration. This indicates that tolfenpyrad inhibits *F. novicida* through a bacteriostatic mechanism.

### *Francisella* species are uniquely sensitive to tolfenpyrad

**FIG 2:**
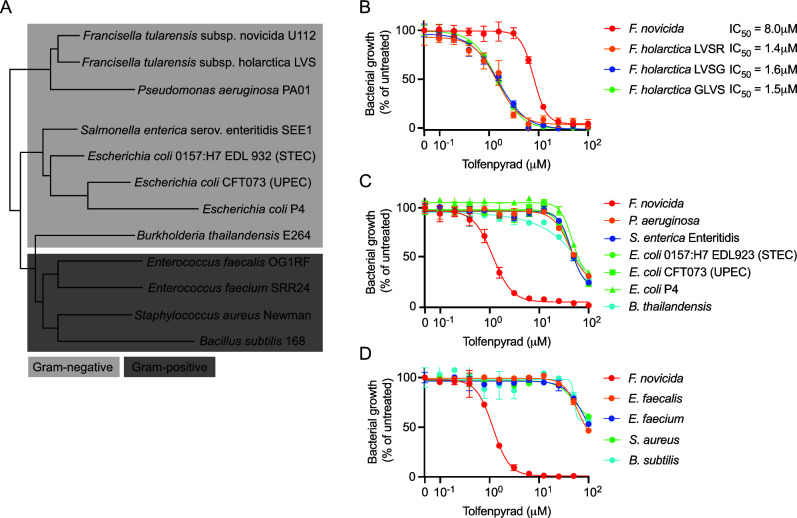
*Francisella* species are uniquely sensitive to tolfenpyrad. (**A**) Phylogenetic tree of bacterial strains included in this study based on their 16S ribosomal DNA sequences. (**B**) Comparison of *F. novicida* to strains of *F. holarctica* in their sensitivity to tolfenpyrad when grown in Mueller-Hinton broth and calculated IC_50_. (**C**) Dose-response curves of *F. novicida* compared to other Gram-negative and (**D**) Gram-positive bacteria grown in tryptic soy broth + 0.1% cysteine.

To characterize the spectrum of activity of tolfenpyrad, we performed *in vitro* dose-response assays on a variety of Gram-negative and Gram-positive pathogens (Fig. 2A). *Francisella novicida* is nonpathogenic to healthy humans but shares 97% nucleotide identity with other mammalian pathogens within the genus, including *F. tularensis* subsp. holarctica (*Francisella holarctica*) (30, 31). Live vaccine strains (LVSs) have been derived from *F. holarctica*, but they retain moderate pathogenicity and yield insufficient protection against the most pathogenic subspecies. We tested the sensitivity of three LVS-derived strains and found that they are greater than fivefold more sensitive to tolfenpyrad than *F. novicida* in Mueller-Hinton broth (MHB) (Fig. 2B). Notably, the IC_50_ of *F. novicida* is considerably higher in MHB because it is not optimal for growth but is permissive for growth of both *F. holarctica* and *F. novicida*. Conversely, tolfenpyrad is >40-fold less potent on other Gram-negative pathogens within Gammaproteobacteria (*Pseudomonas aeruginosa*, *Salmonella enterica*, and *Escherichia coli*) and more distantly related Betaproteobacteria (*Burkholderia thailandensis*) (Fig. 2C). Similarly, tolfenpyrad is considerably less effective on Gram-positive pathogens (Fig. 2D).

The gold standard method for determining antibiotic susceptibilities of pathogenic *Francisella* strains is the broth microdilution method set by the Clinical and Laboratory Standards Institute (CLSI) (32). *Francisella* species and strains are categorized into either Type A or Type B, which are generally more and less pathogenic to humans, respectively. To determine if tolfenpyrad is effective against human-virulent strains of *F. tularensis*, we employed this CLSI method to quantify the minimal inhibitory concentrations of Type A and Type B strains of *F. tularensis*. Indeed, the CLSI-set method revealed that tolfenpyrad inhibits the growth of both types at concentrations varying from 190 to 1,560 nM. When compared to ciprofloxacin, the clinical standard of care antibiotic, the efficacy of tolfenpyrad is 8- to 17-fold less on Type A strains and 2- to 17-fold less on Type B strains (Table 1). The CLSI method confirmed our previous findings by showing that *E. coli*, *P. aeruginosa*, and *Staphylococcus aureus* are insensitive to tolfenpyrad. Since tolfenpyrad inhibits bacterial growth and *Francisella* species are uniquely sensitive, we set out to gain insight into the targeted *Francisella* pathways.

**TABLE 1:** Quantification of the sensitivity of Type A and Type B *Francisella* strains to tolfenpyrad^*a*^

Species	Strain	Type	Ciprofloxacin MIC_50_	Tolfenpyrad	
				MIC_50_	MIC_90_
*F. tularensis*					
subsp. *tularensis*	Schu S4	A	91 nM (0.03 µg/mL)	780 nM (0.3 µg/mL)	780 nM (0.3 µg/mL)
subsp. *tularensis*	MA00-2987	A	91 nM (0.03 µg/mL)	780 nM (0.3 µg/mL)	1,560 nM (0.6 µg/mL)
subsp. *tularensis*	WY96-3418	A	91 nM (0.03 µg/mL)	1,560 nM (0.6 µg/mL)	1,560 nM (0.6 µg/mL)
subsp. *tularensis*	HN63	A	45 nM (0.015 µg/mL)	390 nM (0.15 µg/mL)	780 nM (0.3 µg/mL)
subsp. *holarctica*	CDC-LVS	B	45 nM (0.015 µg/mL)	390 nM (0.15 µg/mL)	780 nM (0.3 µg/mL)
subsp. *holarctica*	KY99-3387	B	91 nM (0.03 µg/mL)	190 nM (0.07 µg/mL)	390 nM (0.15 µg/mL)
subsp. *holarctica*	OR96-0246	B	91 nM (0.03 µg/mL)	390 nM (0.15 µg/mL)	390 nM (0.15 µg/mL)
*S. aureus*	Wichita		1.5 µM (0.5 µg/mL)	>200 µM (>77 µg/mL)	>200 µM (>77 µg/mL)
*P. aeruginosa*	Boston 41501		50 µM (16 µg/mL)	>200 µM (>77 µg/mL)	>200 µM (>77 µg/mL)
*E. coli*	DSM 1103		91 nM (0.03 µg/mL)	>200 µM (>77 µg/mL)	>200 µM (>77 µg/mL)

^*a*^ MIC, minimum inhibitory concentration.

### Identification of genes required for sensitivity to tolfenpyrad

**FIG 3:**
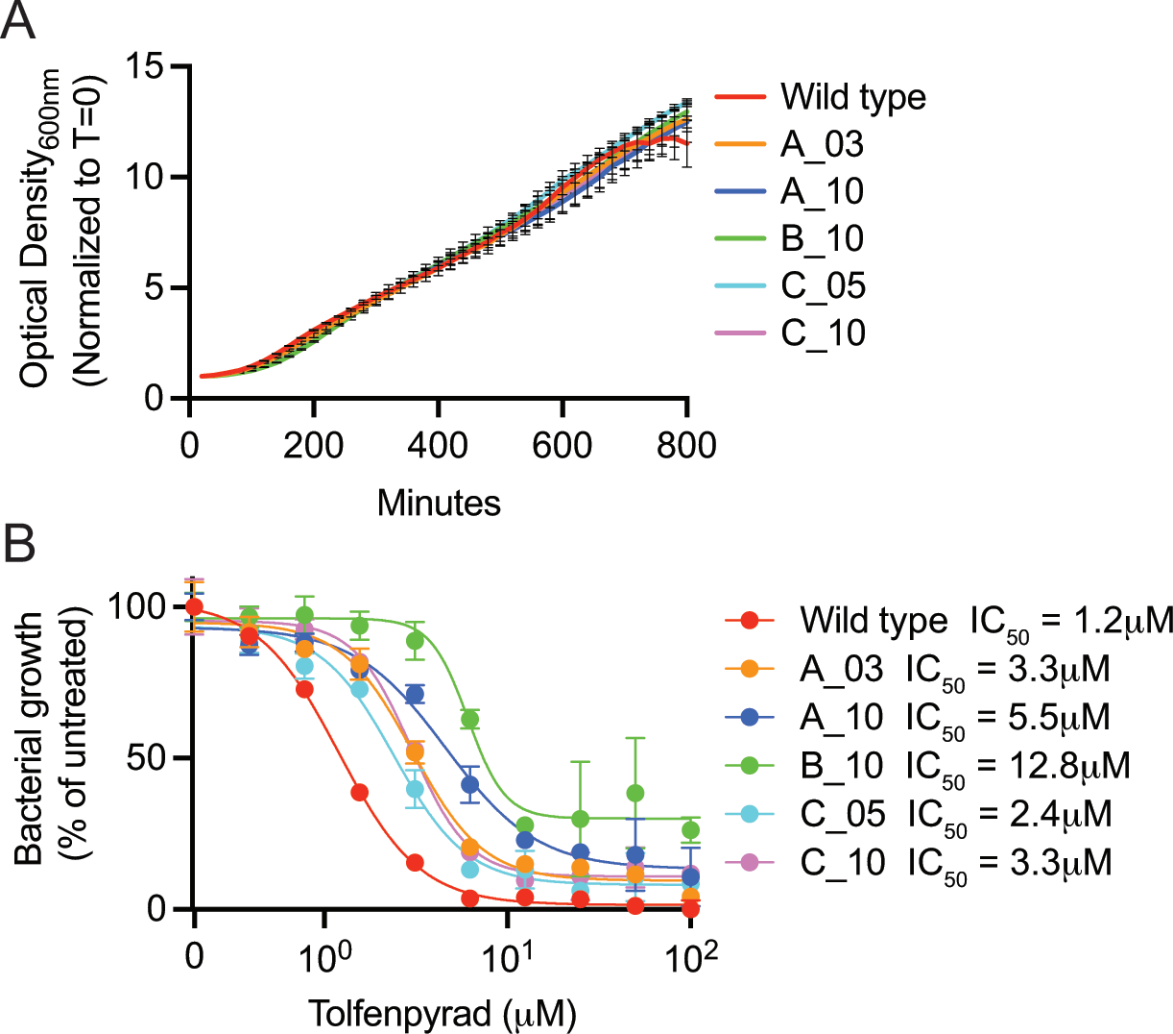
Isolated clones are resistant to tolfenpyrad. (**A**) Growth curves of tolfenpyrad-selected clones, as measured by monitoring optical density at 600 nm in the absence of drug while shaking at 37°C. (**B**) Dose-response curves and approximate IC_50_ values of tolfenpyrad treatment of wild-type *F. novicida* compared to selected clones.

The elucidation of the bacterial pathways targeted by antimicrobials not only yields clues to the drug’s mechanism of action but can also uncover fundamental processes that are essential for bacterial homeostasis. For intracellular pathogens, such as *Francisella*, these pathways often include those that are required for growth *in vivo*. To identify the genes required for sensitivity to tolfenpyrad, we selected a panel of drug-resistant mutants by subjecting 12 independent *F. novicida* cultures to concentrations near the minimum inhibitory concentration (MIC). We chose this relatively high concentration to ensure the selection of spontaneous mutants rather than facilitate adaptation through compensatory mechanisms. After diluting and serially passaging these populations 3, 5, or 10 times in increasing concentrations of tolfenpyrad, we isolated clones on solid agar and characterized them further. Bacteria can tolerate antibiotics through a variety of phenotypic changes, including reduced growth rates. This well-established mechanism of antibiotic resistance is nonspecific, and mutations that result in slow growth are not informative. Thus, we compared the growth rate of the clones to the wild type and omitted those that grew slower from further study. We quantified the resistance profiles of the remaining clones to find five clones that grew at rates similar to wild type and are greater than twofold resistant to tolfenpyrad (Fig. 3A and B). These clones were derived from three independent pools, suggesting that they may contain distinct genetic mutations that lead to resistance.

To identify the mutations acquired during the development of antibiotic resistance, we performed whole-genome sequencing on these five clones and compared the results to wild-type *F. novicida* U112 sequence in the National Center for Biotechnology Information (NCBI; GenBank accession number: CP000439.1). All mutants contained a deletion that resulted in a frameshift in the open reading frame of FTN_0784 (Table 2). We utilized Sanger sequencing to query this part of the genome in our wild-type (parental) strain and found that this mutation is also present in that strain (data not shown). In pool A, the clone selected after 3 days (A_03) contained a point mutation in FTN_1274, an araC/xylS family transcriptional regulator recently termed osrR in *F. holarctica* LVS (33). A second clone from this pool selected after 10 days (A_10) contains this mutation and acquired a second in FTN_1668. This gene, nuoM, encodes the M subunit of NADH dehydrogenase I, which plays a role in respiration. Pool B yielded one resistant clone (B_10) that encodes two mutations. The mutated genes are identical to those in pool A; however, the mutations are distinct, providing further evidence that these genes are important for sensitivity to tolfenpyrad. The two clones selected after 5 and 10 days in pool C (C_05 and C_10) contained an identical mutation in FTN_1274 that was distinct from the others in pools A and B. In total, whole-genome sequencing of the five tolfenpyrad-resistant mutants revealed two mutations in FTN_1668 and three mutations in FTN_1274. These results suggest that nuoM and osrR play a role in sensitivity to tolfenpyrad.

**TABLE 2:** Genetic mutations identified by whole-genome sequencing of tolfenpyrad-selected clones^*a*^

Clone	Genomic position	Mutation	Outcome	Locus ID	Annotation
A_03	842,060	Deletion	Frameshift	FTN_0784	Isochorismatase family protein
	1,346,814	C→T	G54D	FTN_1274	AraC family transcriptional regulator (osrR)
A_10	842,060	Deletion	Frameshift	FTN_0784	Isochorismatase family protein
	1,346,814	C→T	G54D	FTN_1274	AraC family transcriptional regulator (osrR)
	1,783,005	C→T	M252I	FTN_1668	NADH dehydrogenase I, M subunit (nuoM)
B_10	842,060	Deletion	Frameshift	FTN_0784	Isochorismatase family protein
	1,346,773	G→A	P68S	FTN_1274	AraC family transcriptional regulator (osrR)
	1,783,315	G→A	A149V	FTN_1668	NADH dehydrogenase I, M subunit (nuoM)
C_05	842,060	Deletion	Frameshift	FTN_0784	Isochorismatase family protein
	1,346,328	G→A	A216V	FTN_1274	AraC family transcriptional regulator (osrR)
C_10	842,060	Deletion	Frameshift	FTN_0784	Isochorismatase family protein
	1,346,328	G→A	A216V	FTN_1274	AraC family transcriptional regulator (osrR)

^*a*^ Letter refers to the pool, and number refers to the rounds of tolfenpyrad selection.

### NuoM plays an unclear role in controlling sensitivity to tolfenpyrad

**FIG 4:**
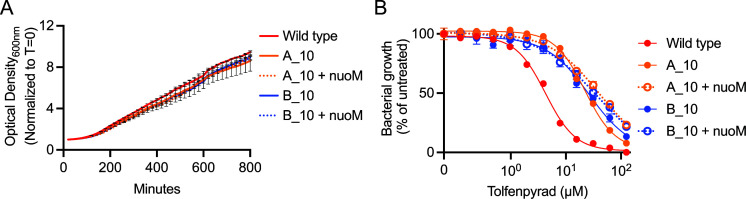
Expression of nuoM in tolfenpyrad-selected clones that carry nuoM mutations does not restore sensitivity. (**A**) Growth curves of wild-type *F. novicida*, tolfenpyrad-selected clones that carry nuoM mutations, and mutants expressing wild-type nuoM, as measured by monitoring optical density at 600 nm in the absence of drug while shaking at 37°C. (**B**) Dose-response curves of the indicated strains, demonstrating that the tolfenpyrad-selected and nuoM-expressing strains are resistant to tolfenpyrad.

NADH dehydrogenase I (NDH-1, Complex I) is composed of 14 subunits and catalyzes the transfer of protons across the inner membrane of Gram-negative bacteria during respiration. NuoM is one of the four proton translocation channels of NDH-1 and is homologous with mitochondrial ND4 in metazoans (34, 35). Since NuoM is functionally conserved between bacteria and metazoans and tolfenpyrad effectively targets both, NuoM may control sensitivity to tolfenpyrad in *Francisella*. To understand the role of NuoM in sensitivity to tolfenpyrad, we set out to delete or mutate FTN_1668; however, after repeated attempts under a variety of conditions, we were unable to make chromosomal changes to nuoM in *F. novicida*. This is not surprising because, although nuoM is not essential in some bacteria, multiple transposon screens in *Francisella* have not recovered transposon mutants in nuoM (36 – 40). To determine if the sensitivity of clones A_10 and B_10, which contain point mutations in nuoM, could be complemented, we used the mini-Tn7 system to constitutively express wild-type nuoM in a heterologous site in the chromosome (41, 42). These complemented strains grew at rates similar to the parental mutants and wild type (Fig. 4A). Expression of wild-type nuoM did not rescue sensitivity to tolfenpyrad as these strains were equally resistant as the parental mutants, indicating that wild-type nuoM does not act in a dominant fashion for complementation (Fig. 4B).

### Point mutations in OsrR confer resistance to tolfenpyrad

**FIG 5:**
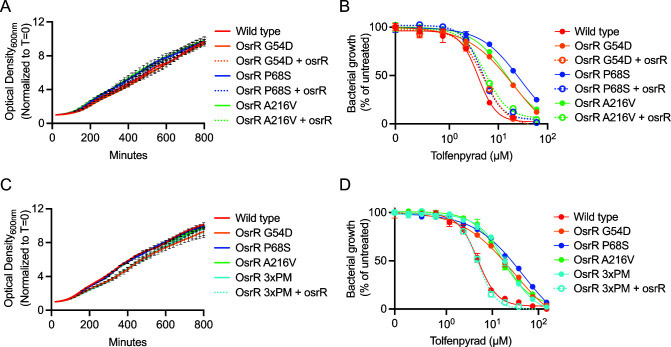
Point mutations in OsrR confer resistance to tolfenpyrad. (**A**) Growth curves of wild-type *F. novicida*, OsrR point mutants (G54D, P68S, and A216V), and mutants expressing wild-type osrR, as measured by monitoring optical density at 600 nm in the absence of drug while shaking at 37°C. (**B**) Dose-response curves of the indicated strains, demonstrating the resistance of the point mutants to tolfenpyrad and complementation of the strains by expressing wild-type osrR. (**C**) Growth curves of wild-type *F. novicida*, OsrR point mutants, a strain encoding all three point mutations (3xPM), and 3xPM expressing wild-type osrR. (**D**) Dose-response curves of the indicated strains.

FTN_1274 is annotated as an “AraC family transcriptional regulator.” The AraC/XylS family represents one of the largest families of bacterial transcriptional regulators and controls various pathways, including virulence and stress response (43). A recent report by Marghani and colleagues demonstrated that the FTN_1274 homolog in *F. holarctica* LVS regulates the primary *Francisella* virulence factor, the T6SS, and the response to oxidative stress *in vitro* (33). Based on these characteristics, they named this gene “oxidative stress response regulator” (osrR). We used allelic exchange in *F. novicida* to engineer strains that encode the three osrR point mutations identified in the genomes of the tolfenpyrad-selected *F. novicida* clones. These in-frame chromosomal mutations were built into the wild-type background to mitigate potential confounding mutations in other genomic loci that are present in the tolfenpyrad-selected clones. Compared to wild type, substitution of glycine for aspartic acid at position 54 (G54D), proline for serine at position 68 (P68S), and alanine for valine at position 216 (A216V) did not affect the growth of the strains (Fig. 5A). In contrast, these strains display four- to sevenfold resistance to tolfenpyrad, mimicking the levels seen in the tolfenpyrad-selected clones (Fig. 5B). Re-expression of wild-type osrR at a heterologous site in the genomes of these point mutants complements the sensitivity of these strains to tolfenpyrad to near wild-type levels. To determine if the mutations work additively or synergistically to cause increased resistance beyond that of a single point mutation, we constructed a strain that simultaneously contains all three mutations in osrR (3xPM). The 3xPM mutant grows similar to wild type, and this strain shares a fourfold resistance to tolfenpyrad, similar to the single-point mutants (Fig. 5C and D). Expression of wild-type osrR in the 3xPM mutant complements sensitivity to tolfenpyrad to parental wild-type *F. novicida*. These data indicate that mutations in any of the three OsrR residues are sufficient for resistance to tolfenpyrad, and that there is no additive or synergistic effect of multiple mutations.

### OsrR is required for growth in IFN-γ treated macrophages

**FIG 6:**
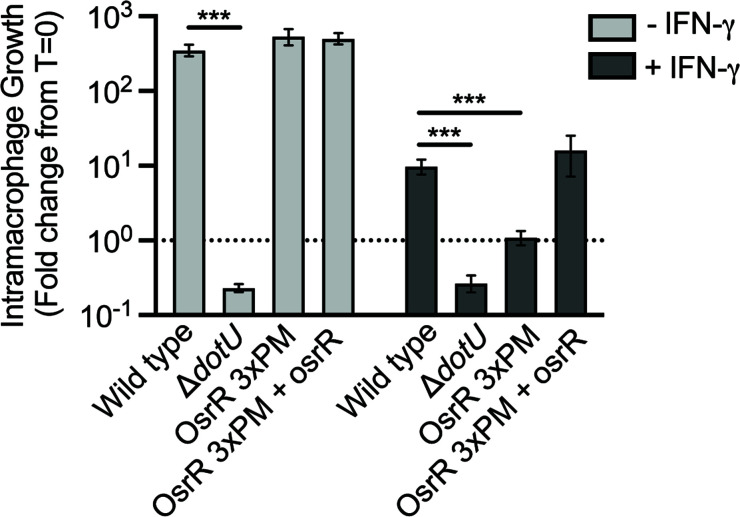
The OsrR point mutant displays attenuated intramacrophage growth in IFNγ-treated macrophages. Growth of wild-type *F. novicida*, an avirulent mutant (Δ*dotU*), a strain encoding all three point mutations (3xPM), and 3xPM expressing wild-type osrR in untreated or IFNγ-treated immortalized murine bone marrow-derived macrophages. Intracellular bacteria were enumerated after 16 h and normalized to the number of bacteria immediately post infection. The y-axis denotes the fold change from *T* = 0. The horizontal dotted line represents no growth over the duration of the experiment, and asterisks denote statistically significant differences from the indicated control. ***P ≤ 0.0001.

Many OsrR homologs that belong to the AraC/XylS family control the expression of virulence factors (43 – 47). Since *F. novicida* is an intracellular pathogen, a common model for virulence is the measurement of bacterial proliferation within cultured phagocytic cell lines. To determine if the OsrR amino acid residues contribute to virulence, we infected immortalized murine bone marrow-derived macrophages and enumerated viable bacteria immediately post infection and after 16 h. Consistent with previous reports, wild-type bacteria grow approximately 400-fold (Fig. 6). The T6SS is required for pathogenesis, and deletion of dotU results in inactivation and death *in vivo*. Compared with wild type, the OsrR 3xPM is not attenuated in naïve macrophages. It is well established that IFN-γ plays a key role in controlling *Francisella* infections *in vivo* (48 – 52). In macrophages, treatment with IFN-γ restricts the cytosolic growth of *Francisella* by inducing a collection of transcriptional responses that program an antiviral and antibacterial state (13). We infected IFN-γ-treated macrophages to test if osrR plays a role in *Francisella* growth within primed macrophages. Wild-type *Francisella* species are significantly attenuated in IFN-γ-treated cells compared to naïve macrophages. Interestingly, the OsrR 3xPM displays 10-fold less growth than wild type in IFN-γ-treated macrophages, and expression of wild-type osrR complements this attenuated phenotype to near wild-type levels. This suggests that OsrR governs the ability of *Francisella* to grow in IFN-γ-primed macrophages; however, the mechanism by which this occurs is unclear.

### Mutation of OsrR increases sensitivity to reactive oxygen species *in vitro* but does not alter expression of type VI secretion system genes *in vivo*

**FIG 7:**
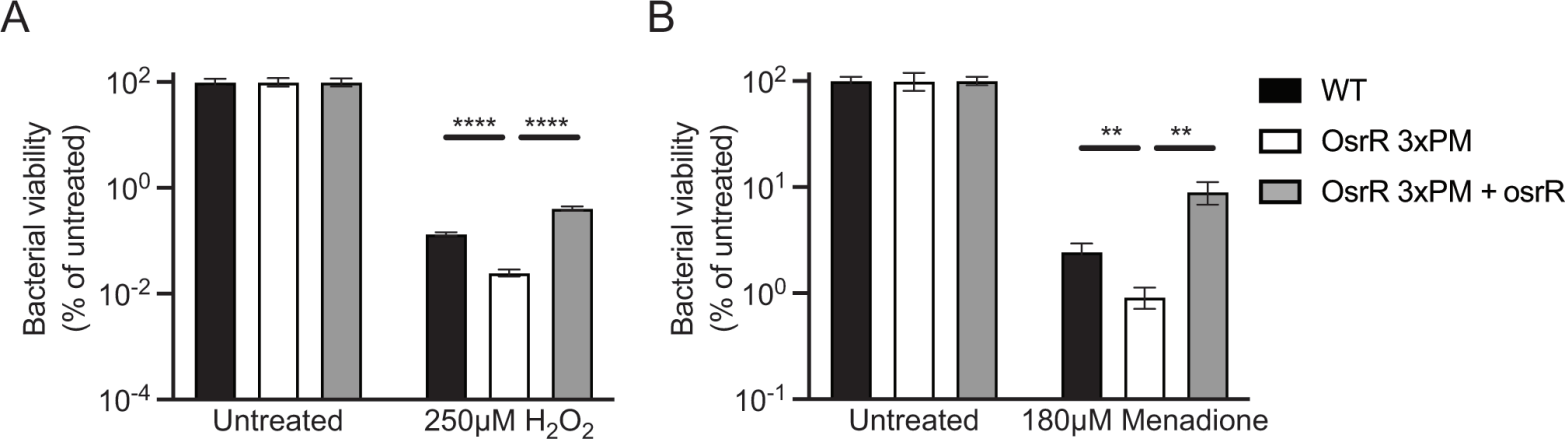
The OsrR point mutant is hypersensitive to reactive oxygen species. Viability of wild-type *F. novicida* (black), a strain encoding all three point mutations (3xPM, white), and 3xPM expressing wild-type osrR (gray) after exposure for 30 min to (**A**) hydrogen peroxide or (**B**) a superoxide-generating drug, menadione. After treatment, the viability of the strains was measured by enumerating CFUs and the untreated groups were set to 100%. **P ≤ 0.01, ****P ≤ 0.0001.

There are at least two potential mechanisms by which an OsrR point mutant may be attenuated in IFN-γ-treated macrophages: (i) mutation of OsrR may block the ability of *Francisella* to withstand the IFN-γ-induced toxic cellular environment, and (ii) OsrR could regulate genes responsible for virulence, such as the type VI secretion system. One of the most prominent functions of IFN-γ is to prime macrophages to respond to intracellular bacteria with oxidative radicals. IFN-γ induces the expression of NADPH oxidase and inducible nitric oxide synthase, enzymes responsible for generating reactive oxygen species (ROS) and reactive nitrogen species, respectively. Deletion of the OsrR homolog in *F. holarctica* LVS causes sensitivity to oxidative stress; thus, we set out to determine if the point mutants identified in this study are hypersensitive to ROS generated by two small molecules, hydrogen peroxide and menadione (33). Under physiological conditions, hydrogen peroxide undergoes the Fenton reaction to generate short-lived (t_1/2_ ~10^−9^ s) ferryl and hydroxyl radicals to induce oxidative stress (53, 54). When treated with hydrogen peroxide, the viability of the OsrR 3xPM strain is fourfold more sensitive than that of the wild type (Fig. 7A). Constitutive expression of the wild-type OsrR in the 3xPM background complements resistance to levels above wild type, suggesting that overexpression may cause resistance above the physiological levels of wild type. Menadione disrupts one-electron redox cycling reactions to reduce molecular oxygen to longer-lived (t_1/2_ = >1 min) superoxide species (55 – 58). Similar to hydrogen peroxide, the OsrR 3xPM strain is hypersensitive to menadione compared to the wild type, and the complemented strain is resistant (Fig. 7B). Based on these results, a functional OsrR protein is required for resistance to oxidative stress, and hypersensitivity of the OsrR 3xPM strain may be a mechanism by which it is attenuated in IFN-γ-treated macrophages.

The *Francisella* T6SS is a well-characterized apparatus that exports toxic proteins directly into host cells and is required for intracellular growth and pathogenicity (59). The T6SS apparatus and a subset of the secreted substrates are encoded in a closely linked cluster of genes called the *Francisella* pathogenicity island (FPI), and additional accessories and substrates are encoded at distal genomic loci (10, 11, 60). Gene expression analysis of a Δ*osrR* mutant in *F. holarctica* LVS incubated with a superoxide-generating drug *in vitro* revealed attenuated FPI gene expression (33). However, it is still unclear if OsrR regulates the expression of FPI-encoded genes *in vivo*. To determine if OsrR regulates the expression of FPI-encoded genes during infection, we performed reverse transcriptase-quantitative PCR on RNA harvested from infected IFN-γ-treated macrophages. IFN-γ induces oxidative conditions and restricts *Francisella* growth once they have trafficked to the cytosol (13). Therefore, we harvested RNA 2 h post infection to allow for the bacteria to be trafficked to the cytosol and exposed to the oxidative conditions but to avoid the bacteria having sufficient time to grow and introduce bias due to strain-specific viability. Expression of a component of the T6SS apparatus (iglA), a secreted substrate (pdpC), and an uncharacterized gene (pdpE) was not altered in the 3xPM or 3xPM expressing wild-type osrR (Fig. S2A through C). Likewise, a mutant that is unable to grow in macrophages (Δ*dotU*) has no difference in the expression of these genes. Since OsrR is likely a transcriptional regulator, there is a formal possibility that it attenuates nuoM expression, and this is a secondary mechanism by which the OsrR point mutant is resistant to tolfenpyrad. However, expression analysis indicates that OsrR does not regulate nuoM expression (Fig. S2D). These studies indicate that while OsrR is not responsible for T6SS gene expression *in vivo*, its function is important to the regulation of sensitivity to oxidative stress.

### Critical OsrR amino acids map to distinct DNA-binding and sensor domains in a structural model

**FIG 8:**
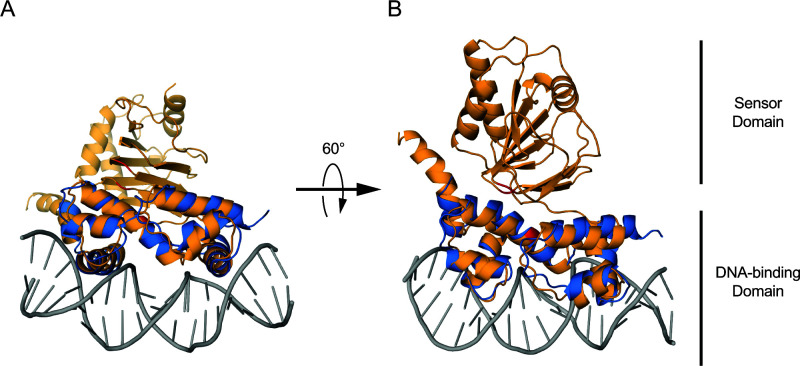
Alignment of a characterized AraC/XylS-family transcriptional regulator, SoxS, with a predicted structural model of OsrR. (**A**) The solved structure of DNA (gray)-bound SoxS (blue, PDB ID: 7W5W) was aligned to an AlphaFold-predicted structure of OsrR (yellow). The OsrR amino acids that were mutated in the 3xPM strain are indicated in red, and the area of structural homology (RMSD = 2.1 Å) is in the foreground. (**B**) The aligned structures are tilted forward 60° to reveal two distinct domains in the predicted structure of OsrR, a candidate sensor domain and a DNA-binding domain.

We explored the functional role that these amino acids might play in OsrR activity by exploring features of its sequence and computationally derived predicted structural models. We utilized the Phyre2 server and AlphaFold to predict the secondary structure of OsrR encoded by *F. novicida* (61, 62). The structural predictions produced by these two tools yielded models that closely aligned with one another, with a root mean square deviation (RMSD) of 2.9 Å (data not shown). To identify the DNA-binding domain of OsrR, we aligned the OsrR model to the Cryo-EM structure of DNA-bound SoxS from *E. coli*, an AraC/XylS-family transcriptional regulator that responds to oxidative stress by binding specific promoters (PDB ID: 7W5W) (63). SoxS aligns to the C-terminal region (residues 163–265) of the OsrR model with a RMSD of 2.1 Å, suggesting that the C-terminal domain is responsible for binding DNA (Fig. 8A and B). Consistent with this hypothesis, the C-terminal domain of OsrR contains two helix-turn-helix motifs (PFAM: HTH_18) that are commonly present in DNA-binding domains of transcriptional regulators and serve to insert into the major groove of DNA for recognition of its binding site (64). Some AraC/XylS-family proteins are composed solely of a DNA-binding domain and interact with distinct sensor proteins for regulatory function. In other members of this family, N-terminal domains play a role in regulation by binding effector molecules to affect gene expression (43, 44, 65, 66). In OsrR, a flexible linker of approximately 12 amino acids connects the C-terminal DNA-binding domain to an N-terminal domain with no significant sequence similarity to characterized AraC/XylS proteins. Since the sequence and predicted structure of the N-terminal domain are unique to OsrR, the identity of a potential ligand is difficult to predict, and the role that it plays in transcriptional regulation is unclear. Interestingly, the amino acid residues that we found to be required for sensitivity to tolfenpyrad and oxidative stress are present in both the N-terminal and C-terminal domains. This suggests that phenotypes caused by the mutations may be caused by two distinct mechanisms. Additionally, although the two mutations in the N-terminal domain are separated by 14 amino acids, the structural model predicts that they are spatially adjacent.

Multiple sequence alignments of OsrR homologs within *Francisella* demonstrate 99% amino acid identity within the genus (Fig. S3A). *Francisella* OsrR diverges from homologs encoded by representative species across Gram-negative and Gram-positive bacterial pathogens with less than 40% identity and 70% similarity; however, even across these diverse species, there are conserved residues in both the N-terminal sensory domains and the C-terminal DNA-binding domains (Fig. S3B and C). The DNA-binding domains of AraC/XylS-family proteins are well-studied, and conserved motifs have been proposed, although from a limited number of homologs (33, 43). The analysis of these homologs reveals an expanded set of conserved residues and shows that two of the conserved residues are among those required for sensitivity to tolfenpyrad and oxidative stress.

## DISCUSSION

*F. tularensis* is one of the most virulent bacterial pathogens in humans. Infection is established with very few bacteria; symptoms are nonspecific, and humans and animals succumb quickly if untreated. Although recent surveys have not identified antibiotic resistance genes in clinical isolates of *Francisella* and effective treatments for tularemia exist, antibiotic-resistant strains can be engineered with little effort, making the discovery of novel therapies essential for public health preparedness (19 – 24). The development of new antibiotics is crucial given the potential for a deliberate release of engineered biological weapons that are antibiotic-resistant. In this study, we utilized a drug repurposing approach by testing a library of antiinfective compounds for their ability to block the growth of *F. novicida*. A series of drugs with antibacterial activity was identified, and among these, an insecticide registered for use in multiple countries, tolfenpyrad, yielded half-maximal inhibition of *F. novicida* growth at a concentration of 1.5 µM (±0.2 µM) (Fig. 1D). Tolfenpyrad exerts a bacteriostatic mechanism of growth inhibition, as concentrations that are 25-fold greater than the IC_50_ inhibit the growth of but do not kill *F. novicida* (Fig. 1E) (67).

The insecticidal activity of tolfenpyrad was first published in 1996. Since then, it has also been shown to be effective against fungi, helminths, mites, ticks, and nematodes (68 – 71). To our knowledge, an antibacterial activity for tolfenpyrad has not previously been found, despite testing of the MMV library against many bacterial species. We demonstrate that *Francisella* species are at least an order of magnitude more sensitive to tolfenpyrad than other bacterial species, possibly providing an explanation why its antibacterial activity has been overlooked in the past (Fig. 2). It is unsurprising that tolfenpyrad is not broadly active against multiple bacterial species, as sublethal exposure of silkworms resulted in the expansion of Enterobacter and Staphylococcus groups among the intestinal microbiota (72). When compared with antibiotics used to treat *Francisella*, tolfenpyrad has a unique chemical structural scaffold, implying that it may represent a potential new class of antibiotics. Structure-activity relationship studies based upon the tolfenpyrad scaffold could potentially increase the potency or broaden the specificity of tolfenpyrad. Such studies have been performed to enhance tolfenpyrad’s activity against eukaryotes, but it is likely that those results are not relevant to *Francisella* because the targets in these organisms are likely to be different. Since tolfenpyrad has been extensively studied and is approved for use in many countries, there exists a multitude of safety and toxicity data obtained for multiple animal models (73). Generally, tolfenpyrad causes little to no toxicity in mammals, depending on dose, route of exposure, and model system. More recently, tolfenpyrad and several derivatives were evaluated for acute toxicity in mice, and it was found that tolfenpyrad has a maximum tolerated dose of 1 mg/kg (74). Further development of tolfenpyrad should include alterations to the tolfenpyrad scaffold to facilitate increased potency and attenuated toxicity, such that the selectivity index is increased prior to development *in vivo*.

A crucial step toward developing tolfenpyrad as an antibiotic is to identify the targeted bacterial pathways. To that end, we generated tolfenpyrad-resistant strains of *F. novicida* and identified multiple genetic changes that confer resistance (Fig. 3; Table 2). Strains that carry mutations in nuoM (FTN_1668) are resistant to tolfenpyrad; however, since nuoM is essential in *Francisella*, we were unable to fulfill molecular Koch’s postulates to establish a causative link between nuoM and sensitivity (Fig. 4). As such, it is unclear if the point mutations in nuoM cause resistance to tolfenpyrad or if the co-occurring mutations in osrR mask the phenotype of the nuoM mutations. However, there is precedent for mutations in NuoM promoting antibiotic tolerance and resistance in *E. coli* and *Pseudomonas aeruginosa*; therefore, it is a formal possibility that NuoM controls sensitivity to tolfenpyrad and NuoM remains a strong candidate for the target of tolfenpyrad (75 – 80). In insects and arthropods, tolfenpyrad inhibits cellular respiration by targeting complex I in the mitochondrial electron transport chain. NuoM is functionally related to and shares homology with eukaryotic ND4, a subunit of complex I. The NuoM-dependent mechanism of resistance may be shared between tolfenpyrad and aminoglycosides. Mutation of NuoM in *E. coli* and *P. aeruginosa* causes resistance to tobramycin-family antibiotics, similar to what we observed for tolfenpyrad in *F. novicida* (76, 77, 79, 80). In addition to the implications for resistance to tolfenpyrad, the mutations that we identified in NuoM do not impact bacterial growth, suggesting that the mechanism underlying tolfenpyrad inhibition of *F. novicida* growth is not dependent on respiration.

We found that three point mutations in a second gene, osrR, confer resistance to tolfenpyrad (Fig. 5B). Furthermore, a strain encoding osrR with all three point mutations was equally resistant to single point mutants (Fig. 5D). This strain displayed attenuated intracellular growth in a macrophage model, but only when the cells were pretreated with IFN-γ (Fig. 6). A report by Marghani and colleagues suggests that OsrR is a transcriptional regulator that modulates expression of genes involved in multidrug efflux, the tricarboxylic acid cycle, the T6SS, the stress response, and other regulatory pathways (33). Since the attenuation we observed was dependent on IFN-γ, we surmised that the most likely explanations are that OsrR either regulates the expression of virulence genes or the ability to persist in the hostile intracellular environment. In our study, we did not observe changes in the expression of genes that comprise the T6SS. There are several potential reasons for this inconsistency with the literature: (i) our expression analysis was performed *in vivo* from bacteria harvested from tissue culture infections compared to bacteria incubated *in vitro* with the ROS-generating drug, menadione; (ii) since we utilized *F. novicida* and the previous research was performed with *F. holarctica*, there may be species-specific differences; and (iii) in our study, we utilized a point mutant compared to deletion of the gene. IFN-γ treatment of macrophages induces ROS and attenuates *Francisella* viability in the cytosol (13). The OsrR point mutant displayed attenuated viability in ROS-generating conditions (Fig. 7). These data provide additional evidence that OsrR plays a role in sensitivity to ROS, and we posit that this sensitivity is the cause of attenuation *in vivo*. Furthermore, this study provides amino acid-level resolution of the physiological activity of OsrR since these experiments utilized a point mutant rather than a deletion of the gene.

The locations of these mutations in the predicted structural model of OsrR map to a sensory domain and a distinct DNA-binding domain (Fig. 8). The two mutations in the sensory domain are 14 amino acids apart but appear to be adjacent to one another in the model. The third mutation is present in the DNA-binding domain, suggesting that the mechanisms by which these mutations have a physiological outcome may be distinct. Alternatively, it is possible that the flexible linker between the two OsrR domains allows the protein to adopt a structure that brings all three of these amino acids within close proximity to each other, and that they participate in the same mechanism. A solved structure with sufficient resolution could answer this unresolved question and would be informative toward understanding the physiology. A survey of OsrR homologs from diverse bacteria suggests that there are conserved residues in both domains. This is particularly surprising for the sensor domains since this family of proteins responds to diverse stimuli.

In summary, we have determined that tolfenpyrad has potent antibacterial activity toward *Francisella* and have used tolfenpyrad as a probe to define the amino acids required for OsrR to regulate IFN-γ-mediated sensitivity to macrophage-produced ROS. Identifying the *Francisella* pathway targeted by tolfenpyrad and characterizing the interaction of the drug with its target will uncover how it exerts genus-specific inhibition. Collectively, these findings are a primer for antibiotic development, characterize the physiological response of *Francisella* to oxidative stress *in vivo*, and elucidate amino acid level details of a transcriptional regulator in *Francisella*.

## MATERIALS AND METHODS

### Bacterial strains and culture conditions

*F. novicida* Utah 112 (U112, NR-13), *F. holarctica* LVSR (capsule-negative variant, NR-597), *F. holarctica* LVSG (NR-585), *F. holarctica* GLVS (Gaisky Live Vaccine Strain 15, NR-14), *F. tularensis* Schu S4 (NR-643), *F. tularensis* MA00-2987 (NR-645), *F. tularensis* WY96-3418 (NR-644), *F. tularensis* HN63 (NR-36146), *F. holarctica* CDC-LVS (NR-646), *F. holarctica* KY99-3387 (NR-647), and *F. holarctica* OR96-0246 (NR-648) from the NIH Biodefense and Emerging Infections Research Resources Repository (BEI resources), NIAID, NIH, *P. aeruginosa* PAO1, and *B. thailandensis* E264 were obtained from Joseph Mougous (University of Washington). *S. enterica* subsp. Enterica serovar Enteritidis, *E. coli* 0157:H7 EDL923 (STEC), and *E. coli* CFT073 (UPEC) were obtained from Subhashinie Kariyawasam (University of Florida). *E. coli* P4 was obtained from Ricardo Chebel (University of Florida). *Enterococcus faecalis* OG1RF, *Enterococcus faecium* ATCC19634, *S. aureus* Newman, and *Bacillus subtilis* MH5636 were obtained from Jose Lemos (University of Florida). *S. aureus* Wichita (29213), *P. aeruginosa* Boston 41501 (27853), and *E. coli* DSM 1103 (25922) were obtained from the American Type Culture Collection. For planktonic bacterial cultures grown for MIC experiments in Table 1, strains were grown in cation-adjusted Mueller-Hinton broth supplemented with 2% Isovitalex (CAMHB) for the IC_50_ calculation that included *F. holarctica* strain in Fig. 2B. Strains were grown in Mueller-Hinton broth supplemented with 0.1% glucose, 2% Isovitalex, and 2.5% fetal bovine serum (MHB), and for the remaining experiments, bacteria were grown in tryptic soy broth + 0.1% cysteine (TSBC). Unless indicated otherwise in the methods, planktonic cultures were grown aerobically at 37°C while shaking. For growth in Petri dishes, TSBC was supplemented with 1.5% tryptic soy agar + 0.1% cysteine and grown at 37°C.

### Screening MMV compounds for inhibitors of *F. novicida* growth

*F. novicida* was grown overnight, diluted, and allowed to grow to OD_600 nm_ = 1.0. For the planktonic assay, compounds were diluted to 25 µM in TSBC and added to a 384-well plate in quadruplicate (*N* = 4/compound). Bacteria were diluted to OD_600 nm_ = 0.1 and added in equal volume to the drug-containing plates. Gas-permeable seals were applied to the top of the plates, and after incubation overnight, turbidity was compared to untreated controls to identify inhibitory drugs. For the solid agar assay, 200 µL of bacteria was spread onto square Petri dishes with sterile glass beads and left to dry. Compounds were diluted to 12.5 µM in TSBC, and 5 μL of the diluted compounds was dropped onto the surface of the agar. After growth overnight, zones of inhibited bacterial growth were noted as inhibitory growth. All of the compounds identified in the primary screen were validated by repeating the assays with the compounds identified in the primary screen (Table S1).

### Calculation of half-maximal inhibitory concentration (IC_50_)

Bacteria were grown to midlog, normalized to OD_600 nm_ = 0.01–0.05, and added to a 96- or 384-well plate containing tolfenpyrad at the indicated concentrations. Plates were incubated at 37°C while shaking in a plate reader, and OD_600 nm_ was measured as frequently as possible to yield a growth curve. For IC_50_ calculation, the OD_600 nm_ of the controls at late log phase was plotted against drug concentration. Controls were set to 100%, and a variable-slope, four-parameter nonlinear regression line was plotted to calculate the IC_50_.

### Mammalian cell culture and intramacrophage growth assay

iBMDMs were obtained from BEI resources and cultured at 37°C with 5% CO_2_ in DMEM supplemented with 10% fetal bovine serum, 4.5 g/L glucose, 2 mM glutamine, 110 mg/L sodium pyruvate, 100 U/mL penicillin, and 100 µg/mL streptomycin. Then, 3 × 10^6^ iBMDM cells were seeded in 24-well plates the day prior to infection in the absence of antibiotic and with 2,000 pg/mL IFN-γ, if indicated. After washing, the cells were exposed to midlog phase *F. novicida* at a multiplicity of infection of 0.1 while centrifuging at 1,000 × *g* for 30 min, followed by 30 min in the 37°C incubator. Cells were washed to remove extracellular bacteria, treated with 50 µg/mL gentamycin, and thoroughly rinsed again before the addition of tolfenpyrad, if indicated. Cells were lysed by the addition of Triton X-100 to 0.1%, and then colony-forming units (CFUs) were enumerated.

### Measurement of tolfenpyrad toxicity on the iBMDM cell line

Then, 4.5 × 10^4^ iBMDM cells were seeded in 96-well plates in the absence of antibiotics and allowed to adhere overnight. On the following day, tolfenpyrad was added to the cells at the indicated concentrations and allowed to incubate for 16 h, and then the MTT assay was performed according to the manufacturer’s protocol.

### Bacterial growth curve with enumeration of CFU

Bacteria were grown overnight, diluted 200-fold, and grown while shaking at 37°C. Samples were collected every 60 min, diluted in TSBC, and plated for enumeration of CFU. After 3 h, tolfenpyrad was added at the indicated concentrations, and samples were collected for CFU enumeration every 30 min until the end of the experiment.

### Minimum inhibitory concentrations

MICs were determined by the microdilution method in 96-well plates according to the Clinical and Laboratory Standards Institute (81). Antibiotics were serially diluted twofold in 50 µL CAMHB. The antibiotic range was 200 to 0.1 µM based on a final well volume of 100 µL after inoculation. The *F. tularensis* inoculum was prepared in CAMHB supplemented with 4% Isovitalex (final concentration in wells, 2%). To each well of the 96-well plate, 50 µL of the adjusted dilution was added for a final inoculum of approximately 5 × 10^4^ CFU/well. Plates were incubated at 35°C. MICs were determined visually at 42–48 h.

### Multiple sequence alignment of 16S rDNA

16S ribosomal DNA sequences for the following bacterial species were downloaded from NCBI: *F. novicida* U112 (NC_008601), *F. holarctica* LVS (NC_007880), *P. aeruginosa* PAO1 (NC_002516), *B. thailandensis* E264 (CP000086), *S. enterica* subsp. Enterica serovar Enteritidis SEE1 (CP011790), *E. coli* CFT073 (CP058222), *E. coli* P4 (DAOJNB010000013), *E. coli* 0157:H7 EDL932 (LPWC02000002), *E. faecalis* OG1RF (CP025020), *E. faecium* SRR24 (NZ_CP038996), *S. aureus* Newman (AP009351), and *B. subtilis* 168 (NC_000964). MUSCLE alignment of these sequences was performed, and a phylogenetic tree was built with the Tamura-Nei genetic distance model.

### Selection of spontaneous tolfenpyrad-resistant *F. novicida* mutants

Twelve independent cultures of wild-type *F. novicida* were diluted 200-fold and grown overnight in the presence of tolfenpyrad at the approximate IC_90_. On the following day, each of the independent cultures was inoculated with a titration of tolfenpyrad and grown overnight. The concentration of tolfenpyrad that restricted growth was selected for subsequent dilution and growth. This process was repeated, and the culture was streaked out for isolation of clonal populations 3, 5, and 10 days after the start of the experiment. Clones that had growth rates similar to the wild type but were resistant to tolfenpyrad were selected for further study.

### Whole-genome sequencing and analysis

Genomic DNA was harvested from bacterial clones using the DNeasy blood and tissue kit (Qiagen) per the manufacturer’s instructions. Sample libraries were prepared by Microbial Genome Sequence Center, LLC, using the Illumina DNA Prep kit and IDT 10 bp UDI indices, and sequenced on an Illumina NextSeq 2000, producing 2× 151 bp reads. Demultiplexing, quality control, and adapter trimming were performed with bcl-convert (v3.9.3). Genomic sequences were aligned and compared to NC_008601.1 using breseq (v0.36.1) to identify variant bases and mutations (82).

### Genetic manipulation of *F. novicida*

Generating in-frame markerless chromosomal changes in *F. novicida* was performed as previously reported (10). Gene expression for complementation in *F. novicida* was performed using the mini-Tn7 system, as previously reported (41).

### Sensitivity of *F. novicida* to ROS-generating compounds

Stationary-phase bacterial cultures were diluted 200-fold and allowed to grow to late log phase, and their concentrations were normalized. Cultures were exposed to the indicated concentrations of H_2_O_2_ and menadione for 30 min, and the CFU from each condition was enumerated.

### Protein structural modeling

The structure of OsrR was predicted by accessing ColabFold and AlphaFold2 through the ChimeraX software (UCSF, version 1.5) (62, 83). Structures were analyzed and displayed using the PyMol Molecular Graphics System (Schrödinger, version 2.5.4). Comparisons of structures were performed using the jFATCAT 2.0 flexible algorithm in the Research Collaboratory for Structural Bioinformatics Protein Data Bank analysis module (RCSB.org) (84, 85).

### Statistical analysis and reproducibility

All data presented in this manuscript are representative of 3 to 11 biological replicates, each of which includes 3 to 8 technical replicates. Graphing and statistical comparison of quantified data were performed by using Prism software (GraphPad, version 9.5.1) to calculate the unpaired *t*-test and *P* values.

## Data Availability

The raw sequence reads of the whole-genome sequences have been deposited into the Sequence Read Archive (SRA) with the following accession numbers: SRX20754764, SRX20754765, SRX20754766, SRX20754767, SRX20754768.

## SUPPLEMENTAL MATERIAL

Fig. S1 spectrum.02713-23-s0001.eps Toxicity of Tolfenpyrad on iBMDM cells.

Fig. S2 spectrum.02713-23-s0002.eps Expression of type VI secretion system genes and nuoM is unaltered in the OsrR 3xPM during macrophage infection.

Fig. S3 spectrum.02713-23-s0003.eps Alignment of the OsrR amino acid sequence with bacterial homologs identifies conserved residues.

Table S1 spectrum.02713-23-s0004.eps Compounds that inhibit the growth of *F. novicida*.

ASM does not own the copyrights to Supplemental Material that may be linked to, or accessed through, an article. The authors have granted ASM a non-exclusive, world-wide license to publish the Supplemental Material files. Please contact the corresponding author directly for reuse.
